# High and Intensive Care in Psychiatry: Validating the HIC Monitor as a Tool for Assessing the Quality of Psychiatric Intensive Care Units

**DOI:** 10.1007/s10488-018-0890-x

**Published:** 2018-08-17

**Authors:** A. L. van Melle, Y. Voskes, H. C. W. de Vet, J. van der Meijs, C. L. Mulder, G. A. M. Widdershoven

**Affiliations:** 1 Department of Medical Humanities, VU University Medical Center, Amsterdam, The Netherlands; 2 GGZ Breburg, Tilburg, The Netherlands; 3 Department of Epidemiology and Biostatistics, VU University Medical Center, Amsterdam, The Netherlands; 4 Parnassia Bavo Group, Rotterdam, The Netherlands; 5 Erasmus Medical Center, Rotterdam, The Netherlands; 6 Department of Medical Humanities, Medical Faculty, VU University Medical Center, F-wing, De Boelelaan 1089a, 1081 HV Amsterdam, The Netherlands

**Keywords:** Models/theories of psychiatry, Quality of care, Validation study, Fidelity scale, Inpatient psychiatry

## Abstract

This study aims to validate the HIC monitor as a model-fidelity scale to the High and Intensive Care (HIC) model, a recently developed model for acute psychiatric wards. To assess the psychometric properties of the HIC monitor, 37 audits were held on closed inpatient wards at 20 psychiatric hospitals in the Netherlands. Interrater reliability, construct validity and content validity were examined. Our results suggest that the HIC monitor has good psychometric properties. It can be used as a tool for assessing the implementation of the HIC model on acute psychiatric wards in the Netherlands, and for quality assessment and improvement.

## Introduction

Quality of care in acute psychiatry is a subject of international debate. There are three main issues of concern: (1) prevention of coercion, especially seclusion (Huckshorn 2006; Noorthoorn et al. 2016; Steinert and Lepping 2009; Voskes et al. 2013); (2) improvement of continuity of care, particularly between in- and outpatient care (Bachrach 1981); and (3) fostering collaboration between mental healthcare professionals, patient, and relatives (Malm et al. 2015). In the Netherlands, patients are generally treated by ambulatory care teams, such as Active Community Treatment teams (ACT), Flexible Active Community Treatment teams (FACT), and by Intensive Home Treatment teams. Admissions to a psychiatric ward can be arranged by these teams, by the police or by psychiatric emergency services. Patients can be admitted to either an open ward or a closed ward in a psychiatric hospital. Currently, the number of beds on closed wards is declining and many open wards have already been closed, thereby increasing the pressure on the remaining wards and the need for quality standards. Over recent years, the High and Intensive Care (HIC) model has been developed to improve the quality of mental health care, specifically inpatient care. Representing a new approach to care, and also new material conditions (van Mierlo et al. 2013), the HIC model has been received with growing enthusiasm. By late 2016, 79% of mental healthcare institutions with closed acute admission wards had adopted it and had joined the HIC foundation to start implementing the model.

Consisting of core interventions and standards for acute inpatient care, the HIC model has been developed through the joint action of professionals, family representatives and peer providers. Its objective is to provide optimal treatment and safety, while restoring and maintaining contact and crisis prevention through a stepped-care principle that combines the medical and the recovery models of care. Key elements of the model are emphasis on collaboration with outpatient care, patients and relatives, a comprehensive admission process, and a healing environment. In the HIC model, a “high-care-function” and an “intensive-care function” are combined. Initially, patients are admitted on the High Care section (HC). In case stress, anxiety and agitation rise, or when aggression is imminent, one-to-one care can be given at the HC, or depending on the severity and nature of the crisis patients can go (accompanied by a nurse of the HC), to the Intensive Care Unit (ICU). Attached to the ICU there can be a High Security Room (HSR), the last of which is to be used only as a last resort. Although the design of the ICUs follows that of many PICUs as found in the UK and Scandinavian countries, a mayor difference is the close connection of the ICU section with the general HC ward, and thus avoiding often disrupting transport of patients to other units in case of severe disruptive behaviour (Bowers et al. 2008; Vaaler et al. 2006). The aim of the ICU is to be able to provide one-to-one supervision in a separate space to patients who cannot stay with the other patients on the HC and to avoid seclusion in a HSR, thereby improving the quality of care.

In terms of professional practice and as a set of material conditions, the development and implementation of the HIC model involves major inputs by mental healthcare institutions. To monitor these inputs and to generate and maintain motivation for implementing the model, a trustworthy model-fidelity scale is needed. Since the model is multifaceted—comprising various components, each important to the quality of HIC—we wanted to be able to measure the extent to which the model has been implemented. To meet these purposes, the HIC model-fidelity scale—named the HIC monitor—was developed on the basis of literature research and expert consensus (Table 1).

**Table 1:** Examples of the HIC monitor items

Item	Score 1	Score 2	Score 3	Score 4	Score 5
Small caseload: day shift: the ward has an optimal ratio of 7 nurses per 20 beds	3 nurses or fewer per 20 beds	4 nurses per 20 beds	5 nurses per 20 beds	6 nurses per 20 beds	7 nurses or more per 20 beds
Coordination of care meeting (CCM): a meeting to establish treatment goals. It is held at admission	No CCM takes place within 24 h of admission	Under 25% of patients get a CCM within the first 24 h of admission	25–49% of patients get a CCM within the first 24 h of admission	50–74% of patients get a CCM within the first 24 h of admission	75–100% of patients get a CCM within the first 24 h of admission
Healing environment (HE): the ward uses an instrument (such as OAZIS) to assess and improve the level of HE	Little to no attention is paid to the living space or environment	The environment is adequate but there is no HE policy	Although the ward is familiar with principles of HE, this is not tangible in the environment	HE is a structural focus of attention, but it is not assessed	HE is a structural focus of attention, whose level is regularly assessed and improved

Using a model-fidelity scale to assess the quality of a mental health services model has several benefits (Vugt et al. 2011). As well as providing insight into the level of implementation of the specific model, scoring shows the extent to which components of the model have been implemented, and thereby creates a basis for future research on the model’s effects, such as research on the quality of care or on reductions in coercion. And as well as providing targets for improving the services provided, the HIC monitor scores can also be used for benchmarking purposes.

However, in order for the HIC monitor to be valuable, it needs to be validated. This means establishing inter-rater reliability, content validity and construct validity. Firstly, inter-rater reliability involved the congruence between scores of various people who use the monitor. Secondly, content validity required analysis of the extent to which the items of the monitor reflected the content of the model (Mook 2001). Acting on the principles outlined by Burns and Grove (1993), we therefore draw on three sources of information: literature research, expert consensus and surveys of the experiences of staff and auditors. During the development of the HIC monitor, the former two sources were used to foster content validity. In this study content validity was further established by focusing on the latter. Lastly, construct validity concerned the HIC monitor’s ability to distinguish between various levels of the model’s implementation (Cronbach and Mehl 1955). To assess it, we investigated whether the score on the monitor reflected the HIC model’s level of implementation in psychiatric wards.

## Methods

### Instruments

#### HIC-Monitor

The first version of the HIC monitor consisted of 50 items divided over 11 domains. The domains were (I) team structure, (II) team processes, (III) diagnostics, treatment, and treatment interventions, (IV) organization of care, (V) monitoring, (VI) professionalization, (VII) the Psychiatric Hospitals Compulsory Admissions Act (BOPZ), (VIII) the electronic health record, (IX) healing environment, (X) safety; and (XI) evaluation of and feedback on coercion. A separate score sheet allowed an acute admission ward to be scored according to the criteria of the HIC monitor. Scoring was done on the basis of a five-point scale (1–5) in ascending order from “not implemented” to “fully implemented”. Five items were scored on a three-point scale that assigned scores of one, three and five. Items referring to the presence of ward facilities were scored dichotomously. Scoring in the HIC monitor was intended to assess the current situation, not projected plans or goals.

### Procedures

#### Sample of Wards

Data was collected on closed acute admission wards for adult psychiatric patients (aged 18 and older) in various mental healthcare institutions in the Netherlands. Patients admitted to these wards were in acute psychiatric crisis situations, many of whom were admitted involuntarily. The participating mental healthcare institutions all provided both inpatient and outpatient services. Although some larger mental healthcare institutions with bigger catchment areas have multiple acute admission wards, no other separate intensive inpatient units in acute psychiatric care for adults, such as PICU, exist in the Netherlands. The selection of wards was done by mental healthcare institutions that participated in the development and implementation of the HIC model. Each participating institution was asked to select two acute closed wards for adult patients in which they could implement the HIC model. As institutions implemented the HIC model at different times and in different phases, levels of implementation also differed.

#### Training of Auditors

Data was collected in audit visits, for which 26 auditors were recruited by inviting each institution to provide one or more staff member from the participating wards. A 1-day training program was organized for all auditors. During the research period, three follow-up meetings were organized for the auditors to exchange experiences and to further improve the uniformity of the audit process.

#### Audits

Per audit, two auditors visited the ward simultaneously. Before the audit, the manager of the ward had used a questionnaire to collect basic information on team structure and the organization of care. At the ward, the auditors observed a multidisciplinary meeting in which staff discussed care for individual patients. They then interviewed nurses, medical staff, managers and one patient, and used a checklist to examine the health records. After the audit, each auditor *independently* filled in the score sheet for the HIC monitor, and sent it to the researchers. To ensure that inter-rater reliability was assessed correctly, the two auditors were not allowed to discuss the scores they gave.

In a focus-group discussion with the health care professionals at each ward, the researcher (LvM) gave feedback on the auditor’s independent monitor scores. The discussion had a dual purpose. The first was to use the feedback on the scores as the basis for internal evaluation. The second—useful for research purposes—was to ensure both that the interpretations of the scores and that the auditors’ and professionals’ experiences provided insight into the relevance, comprehensiveness and comprehensibility of the HIC monitor and the auditing process. The focus-group discussions involved ward managers, psychiatrists and nurses (and, if available, peer experts, nurse specialists and psychologists), and were organized on all participating wards. To allow data-analysis, the discussions were recorded.

### Assessment of Reliability and Validity

#### Inter-rater Reliability

To assess the inter-rater reliability per item, we examined the average agreement of the auditors’ scores. Percentages of corresponding scores were used as measure of agreement (Kottner et al. 2011). Per item, we calculated the percentages of corresponding scores and corresponding scores, allowing for a one-point difference (Kottner et al. 2011). As domain scores of the HIC monitor might be used in future applications, we compared the average scores awarded by both auditors per domain. The SD of the mean differences (paired *t* test) indicates the agreement in scores.

#### Content Validity

To analyze the content validity of the HIC monitor in terms of relevance, comprehensiveness and comprehensibility (De Vet et al. 2011), we analyzed (1) the auditors’ reflections during the follow-up meetings, and (2) the outcomes of the focus-group meetings in the institutions. Items were altered in response to the feedback given in both types of meeting, and items that consistently scored low or high, thereby reducing their ability to distinguish model-fidelity standards.

#### Construct Validity

To examine the construct validity, we formulated a hypothesis regarding any relation between a participant institution’s level of implementation of the HIC model and its scores on the HIC monitor. The hypothesis was that the score on the HIC monitor would be higher at institutions that had been involved in the development of the HIC model from the beginning (and had thus started implementing HIC before the start of the study) than at institutions that had not (and had therefore planned or begun to implement the model only at the start of the study). To test this hypothesis, the participating institutions were divided into two groups. The first consisted of 11 institutions that were expected to score higher on the HIC monitor because they were early adopters. The other consisted of ten institutions that were expected to score lower on the HIC monitor because they were either relatively late to implement the HIC model or were just starting to implement it. A *t* test for independent samples was used to compare the mean scores of these two groups on the HIC monitor.

All analyses were performed in SPSS version 22 (IBM Corp., Armonk, NY).

## Results

### Sample Characteristics

Twenty-five large mental healthcare institutions in the Netherlands were asked to participate in this study. Twenty-one (84%) agreed, representing 79% of the total number of closed beds in acute psychiatric hospitals in the Netherlands. One institution was excluded, as it offered to participate with a single ward that specialized in addiction care. One ward at another institution was excluded from analysis, as it turned out to specialize in long-stay care rather than acute care. As 17 institutions participated with two wards, and three participated with one ward, the 20 institutions included represented a total of 37 wards. Twelve of these institutions provided one auditor, and five provided more than one. The audit team consisted of nurses, managers, psychiatrists, and policy officers. All auditors had clinical or managerial experience with acute psychiatric care.

### Inter-rater Reliability

Table 2 presents the agreement percentages per item of the two independent audit scores. For all items, it shows the percentages of exact agreement, and the percentages of agreement when a one-point difference in scores was allowed. The percentages for exact agreement show that 52 items scored below the threshold of 75% agreement. When a one-point difference was allowed between audit scores, 12 items scored below the 75%, thereby obtaining a relatively good agreement for most items. When a one-point difference in scores was allowed for the two items with the lowest exact agreement percentages—“somatic screening during admission” (28.57%) and “electronic health record” (28.57%)—the respective agreement percentages increased to 80 and 62.86%. On the same basis, the items with the lowest agreement percentages were “partnership agreement on safety” (57.14%) and “evaluation of coercion” (57.14%). Due to the low percentage, the former item (“partnership agreement on safety”) was dropped. In view of the importance to the model of evaluating coercion, “evaluation of coercion” was reformulated as two separate items, the first stressing the evaluation of coercion within the team to adjust future actions, and the second focusing on the evaluation of coercion with the patient and relatives. For the domain scores, the standard deviation of the mean difference between two auditors was < 0.9.

**Table 2:** Inter-rater reliability of audit scores and average audit scores of the HIC monitor (N = 37)

Item	Average score (SD)	Audit score	
		% Exact agreement	% Agreement if 1-point difference allowed*
Team structure			
Small caseload: day shift (1a)	3.12 (1.22)	65.71	88.57
Small caseload: evening shift (1b)	2.96 (1.25)	80.00	94.29
Small caseload: night shift (1c)	2.33 (1.08)	74.29	85.71
Stepped care (2)	3.58 (1.47)	71.43	94.29
Staff coverage (3)	4.50 (0.64)	71.43	94.29
Team (4)	2.61 (1.64)	40.00	60.00
Psychiatrists (5)	2.78 (1.46)	51.43	65.71
Psychologists (6)	1.30 (0.78)	85.71	97.14
Nursing specialists (7)	1.96 (1.47)	68.57	80.00
Nurses/SPH (8)	1.99 (1.49)	82.86	91.43
Addiction experts (9)	1.71 (1.51)	85.71	88.57
Peer providers (10)	1.41 (0.91)	82.86	97.14
Activity supervisors: FTE (11a)	2.75 (1.44)	62.86	85.71
Activity program (11b)	2.58 (1.01)	51.43	97.14
Supervisors/team leaders (12)	3.25 (1.16)	31.43	77.14
Extra disciplines (13)	3.54 (1.09)	48.57	94.29
Team processes			
Vision/work methods (14)^~^	2.55 (1.41)	68.57	
Hospitability and presence (15)	3.07 (1.05)	37.14	77.14
Attitude/treatment (16)	2.64 (1.25)	37.14	77.14
Coordination of care meeting: at admission (17a)	2.09 (1.29)	48.57	77.14
Coordination of care meeting: every 3 weeks (17b)	3.07 (1.75)	45.71	77.14
Coordination of care meeting: at discharge (17c)	2.93 (1.56)	37.14	71.43
Digital whiteboard (18)	1.99 (1.45)	74.29	91.43
Care process and consultation: HIC (19a)	3.13 (1.85)	51.43	65.71
Care process and consultation: ICU (19b)	1.80 (1.51)	68.57	77.14
Care process and consultation: HSR (19c)	1.71 (1.40)	60.00	68.57
Diagnostics, treatment, and treatment interventions			
Guidelines (20)	3.50 (1.36)	42.86	65.71
Early diagnostics at admission (21)	4.26 (1.27)	51.43	80.00
Copy of treatment plan (22)	2.32 (1.60)	62.86	88.57
General examination: history (23a)	2.91 (1.46)	37.14	85.71
General examination: medical (23b)	3.95 (1.43)	54.29	71.43
Risk assessment (24)	2.43 (1.52)	60.00	88.57
Conflict control and personal safety (25)^~^	4.26 (1.34)	65.71	
Early and emergency medication (26)	3.53 (1.40)	62.96	77.14
Psycho-education (27)	2.54 (1.04)	37.14	74.29
Somatic screening during admission (28)	3.57 (1.01)	28.57	80.00
Dual diagnosis (29)	1.71 (1.02)	57.14	82.86
Family interventions (30)	3.18 (1.04)	51.43	82.86
Organization of care			
Admission and discharge (31)^~^	3.13 (1.71)	54.29	
Waiting list (32)	4.53 (0.93)	85.71	100
Monitoring			
ROM (33)	2.05 (1.61)	71.43	80.00
ROM usage (34)	1.63 (0.99)	74.29	94.29
HIC improvement-curve (35)	2.79 (1.39)	42.86	77.14
Professionalization			
Reflection (36)	3.22 (1.79)	60.00	88.57
Education (37)	3.07 (1.72)	54.29	68.57
Knowledge of FACT/ambulatory care (38)^~^	3.58 (1.21)	57.14	
Team spirit (39)	3.93 (1.06)	45.71	80.00
Psychiatric Hospitals Compulsory Admissions ACT			
Execution of Psychiatric Hospitals Compulsory Admissions ACT (40)	4.11 (0.87)	54.29	94.29
Electronic health record			
Electronic health record (41)	3.53 (1.38)	28.57	62.86
Healing environment			
Healing environment: HE (42)	2.57 (1.20)	57.14	85.71
HC: individual rooms and bathrooms (43a)^~~^	51.35%	91.43	
HC: comfort room (43b)^~~^	56.76%	85.71	
HC: diversity of meeting spaces (43c)^~~^	70.27%	80.00	
HC: outside space (43d)^~~^	94.59%	91.43	
HC: family room (43e)^~~^	27.03%	82.86	
HC: time-out/emergency bed (43f)~~	37.84%	74.29	
HC: open workspace (43g)^~~^	24.32%	97.14	
HC: domotics (43h)^~~^	27.03%	74.29	
IC (44)	2.29 (1.58)	62.86	80.00
ICU (45)	2.14 (1.48)	71.43	85.71
High Security Room (46)^~^	1.53 (1.19)	91.43	
Safety			
Safety-management system (47)	4.05 (4.05)	65.71	97.14
Partnership agreement on safety (48)	3.72 (3.72)	45.71	57.14
Evaluation of and feedback on coercion			
Evaluation of coercion (49)	3.16 (1.35)	45.71	57.14
Argus (50)	3.17 (1.45)	51.43	85.71
Total average score	2.92 (0.84)		

*For the items scored on a 5-point Likert scale, “agreement” was extended to include scores that differ by 1 point on both measurements

^~^Items scored trichotomously (1, 3 and 5)

^~~^Items scored dichotomously (% yes)

### Content Validity of the HIC Monitor

It was shown by analysis of the auditors’ reflections during the follow-up meetings and of the participants’ interpretations and experiences in the institutions’ focus-group meetings that the comprehensiveness and comprehensibility of the HIC monitor were satisfactory. The HIC monitor also appeared to be a useful tool in audits and focus groups.

Further analysis of the content validity consisted of two steps. The first involved calculating the low- and high-scoring items. While high-scoring items might indicate a high general standard, suggesting that no improvement is needed, low-scoring items might indicate criteria that have either been set too high or have not gained priority in the implementation process. Table 2 shows an overview of the average item scores broken down by domain. The average score across all items was 2.92. The lowest mean scores were found in the team-structure domain, whose lowest-scoring item was “the presence of a psychologist” (1.3). Other low-scoring items in this domain were “the presence of an addiction specialist” (1.41), “the presence of a peer provider” (1.72), and “the presence of a nurse practitioner” (1.96). In the remaining domains, low scores on the items scored on a five-point scale were found for “performing Routine Outcome Measurement” (1.63), “providing dual diagnosis treatment” (1.71) and “having a digital whiteboard” (1.99). High scores on the items scored on a five-points scale were found for “team spirit” (3.93), “safety-management systems” (4.05), “Execution of Psychiatric Hospitals Compulsory Admissions ACT” (4.11) and “early diagnostics at admission” (4.26).

In the second step, the comments on the meaning of the lowest and highest-scoring items for the HIC model were discussed to decide whether or not the item should be kept. It appeared that some of the lower averages may have been caused by unclear definitions and by the way the items were phrased. These were reasons to change the formulation. For example, the term “legal consultant” was replaced by “patient representative”, and the term “domotics” was replaced by “electronic support”.

Due to the feedback given during focus group discussions at the wards and follow-up meetings with the auditors, new standards were added in seven items of various domains, and one new item, “transition to outpatient care”, was added to the “organization of care” domain. In 12 items, criteria were revised. In the item “education”, six criteria for staff education were added: early risk assessment, family interventions, psychopathology, psycho-pharmaca, suicide prevention and observational techniques. The low-scoring item “electronic health record” was removed because the content of the item did not fit the HIC model. According to their content and purpose, several items were moved to other domains to better correspond to the essence of the domain. For example, “treatment plan” was moved from the “diagnostics, treatment, and treatment interventions” domain to the “team processes” domain, where it was closer to the item “coordination of care meeting” in which the treatment plan is made.

To ensure face validity and comprehensibility of items of the final version, adjustments to the HIC monitor were checked with the auditors during the last follow-up meeting.

### Construct Validity

**Fig. 1:**
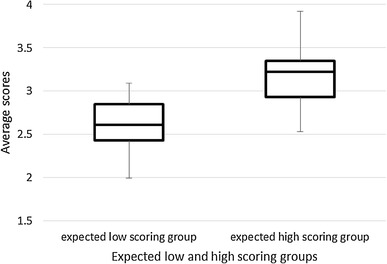
Scores of expected low and high scoring groups

The data supported our prediction that the institutions that had been involved in the development and early implementation of the HIC model would score higher on the HIC monitor than those that had just started to implement it (Fig. 1). The wards in the group we had expected to score higher on the HIC monitor scored a mean value of 3.18 (SE = 0.08) versus 2.60 (SE = 0.07) for the wards in the group we had expected to score lower. This difference was statistically significant (*p* = < 0.001).

## Discussion

### Main Findings

Our results show that the HIC monitor has a reasonably good inter-rater reliability and satisfactory content and construct validity.

Although Cohen’s Kappa, a relative measure of reliability (Cohen 1960), is a well-known measure of agreement between two nominal or ordinal variables for calculating inter-rater reliability, an absolute measure of agreement is much more informative (De Vet et al. 2013). We therefore chose to use agreement percentages as a measure of inter-rater reliability. Some items were not scored on a 1–5 Likert scale, but had only 2 or 3 response options, which made it easier to achieve agreement. The items that scored lower than an arbitrary cutoff point of 75% agreement were revised. A possible explanation for these lower percentages in agreement is that the item was not easily comprehensible, and that its purpose was therefore unclear. The combination of agreement percentages with extensive feedback on the HIC monitor allowed the lower scoring items such as “team” and “evaluation of coercion” to be further refined.

Our assessment of the content validity of the HIC monitor concerned evaluation of the relevance, the comprehensiveness and comprehensibility of the items. This led us first to eliminate several original items that the auditors and professionals considered to be irrelevant to the HIC model. Next, to achieve the primary function of the HIC monitor—to comprehensively indicate the extent to which the HIC model had been implemented—we added the item “transition to outpatient care” to the original items. Insight into the comprehensibility of the HIC monitor was provided by the interpretations and experiences of professionals in the institutions. The HIC monitor also appeared to be a useful tool in audits and focus groups, which also tended to confirm its content validity. Further evidence of the monitor’s good content validity is provided by the fact that no other aspects of the HIC model had been missed by the stakeholders in the research. Lastly, to enhance the comprehensibility of the HIC monitor, we reformulated some items on the basis of the feedback provided in the focus group discussions and by the auditors. Thus, while changes to the HIC monitor were limited, maximum improvement was reached in clarifying the content of items.

We retained a number of items which had attained the maximum score by either very low numbers or very high numbers of wards. This was primarily because stakeholders in the focus groups described these items as important components of the HIC model. A second reason was that some of the 37 participating wards showed that it was possible to meet the criteria, thus indicating that implementation of the respective items in practice was feasible. Although some domains contained a limited number of items and one domain contained one item, the current domain structure was maintained. The reason for this was that the content of the HIC monitor should reflect and encompass the entire HIC model, even if this meant that several items and domains would contain a low number of items. Moving items to other domains would therefore be artificial and would undermine the coherence and comprehensibility of the other domains.

Regarding the construct validity, our results showed that the HIC monitor can distinguish between the two groups of institutions, thereby demonstrating a measure of the level of implementation of the HIC model. As there are no other instruments to measure this level of implementation, this was the only way to obtain construct validity. As far as we know, the HIC monitor is the first instrument to assess implementation of a model for acute psychiatric wards. This means that there is no gold standard with which it can be compared.

### Strengths and Limitations

This was the first study intended to validate an instrument for assessing the quality of implementation of the HIC model, a new model of care in acute psychiatric hospitals. One particular strength is the fact that all 37 wards—in itself a high number—were assessed by two independent and trained raters. Another is that this work resulted in a HIC monitor with satisfactory psychometric properties.

The study had three main limitations. First, the wards where audits took place were selected by the participating mental healthcare institutions. Since the institutions differed in terms of the number of wards and of the extent to which the HIC model had been developed, they may have selected wards on which the implementation of the HIC model was best established, thus leaving worse performing wards out of the picture. If so, this might have given a more positive view of the development of the HIC model within those institutions. This does not affect the validation of the HIC monitor, even though both early and late implementing institutions may have chosen their best wards.

The second limitation is that, although we were able to determine the content and construct validity, we could not assess the criterion validity, as there is no gold standard for the quality of psychiatric intensive care units. In the future, one might consider examining the relationship between scores in the HIC monitor and outcomes for HIC wards, such as any reduction in the use of coercive measures, any reduction in the length of hospital stay, and any improvement in the quality of care. This establishment of criterion validity, in terms of predictive validity, can be seen as an essential step towards determining the practical utility of a model-fidelity scale (Donabedian 1966; Lloyd-Evans et al. 2016). The HIC monitor can lead to improved quality of care by (1) making transparent the current quality of care of the acute admission ward, (2) providing opportunities for step by step improvement of quality of care by addressing specific aspects of care which have not been adequately implemented according to the scores on the HIC monitor, and (3) providing an opportunity for certification of “good quality acute admission wards”, using cut-off levels of the HIC monitor total scores, creating a basis for a possible new gold standard. However, any demonstration of the relationship between criteria and the intended outcome should take account of the process in which the model-fidelity scale was developed. As the HIC monitor was created through expert consensus and contains a collection of best and evidence-based practices, it can best be described as “a sum of its parts”—which makes individual analysis of its components less relevant.

The third limitation is that, to optimize the monitor’s content validity, some final adjustments were made to its content. In one sense this is a strength of the study: the adjustments to the instrument were based on the auditors’ feedback and on the focus group discussions with the mental healthcare institutions. The drawback is that the HIC monitor was adapted during the evaluation process integral to this study—the practical implications being that the adjustments made to the HIC monitor should be tested in practice and that further refinement of the items might prove to be necessary at a later stage.

### Future Research

The HIC monitor can now be used in future studies assessing the implementation of the HIC model. Our results have already led the HIC monitor to be provided to psychiatric hospitals for use as a means of improving the implementation of the HIC model. Further research could focus on the associations between the HIC model and development of frameworks for outcome parameters such as patient satisfaction with the quality of care, length of stay, the number of aggression incidents and coercive measures. Currently, we are undertaking research on quality of care perceived by patients using the KWAZOP, a Dutch instrument to assess the quality of care on closed psychiatric admission wards, and on the use of coercive measures at HIC wards and will compare this to HIC monitor scores (Nijssen et al. 2001). A second topic of research might concern the feasibility and practical usability of the HIC monitor in implementing the HIC model and in using the HIC monitor as a tool for identifying aspects of the ward that are in need of improvement. A possible third option would be an international comparison of the HIC model to the provision of psychiatric intensive care units (PICU’s) in other European countries.

To increase the HIC monitor’s utility and feasibility, we ensured a uniform external audit method by using both interview and scoring guidelines and checklists. A structure for the audit was also provided that could facilitate standardization scoring of the HIC monitor. Although an audit was a fairly time-consuming means of scoring the HIC monitor (1 day for assessment), we have shown that it is possible to use the HIC monitor on a relatively large scale. However, we could not further explore whether the construction of the monitor—which includes an item-by-item explanation—would enable a valid and reliable internal audit.

## Conclusion

In conclusion, as a useful tool for assessing the level of implementation of the HIC model on acute psychiatric wards, the HIC monitor can be used for quality assessment and improvement. Our study shows that the HIC monitor has reasonably good psychometric properties. Due to the consensus that was sought during its development and validation, it is an instrument that corresponds closely to daily practice, and may thus benefit the implementation of the HIC model on acute psychiatric wards. As it can be used to study the associations between the components and outcomes of the HIC model (use of coercion, patient satisfaction), it can contribute to the improvement of quality of care for acute psychiatric patients.

## Data Availability

Data supporting the findings of our study can be found in Table 2. The final HIC monitor is available in Dutch at http://hic-psy.nl/wp-content/uploads/2016/04/HIC-monitor.pdf. We encourage the reader to contact us in case of any questions or need for translation.
